# Supplementary material for: Subcellular Localization of uc.8+ as a Prognostic Biomarker in Bladder Cancer Tissue
Source: Cancers (Basel). 2021 Feb 8;13(4):681. doi: 10.3390/cancers13040681 (PMC7914980; doi:10.3390/cancers13040681)
Supplement: Supplementary file 1 [file cancers-13-00681-s001.pdf]

# Subcellular localization of uc.8+ as a prognostic biomarker in bladder cancer tissue

**Sara Terreri [a, b, 1], Sara Mancinelli [a, c, 1], Matteo Ferro [d, 1], Maria Concetta Vitale [e], Sisto Perdonà [f], Luigi Castaldo [f], Vincenzo Gigantino [g], Vincenzo Mercadante [a], Rossella De Cecio [g], Gabriella Aquino [g], Marco Montella [h], Claudia Angelini [i], Eugenio Del Prete [i], Marianna Aprile [a], Angelo Ciaramella [e], Giovanna L. Liguori [a], Valerio Costa [a], George A. Calin [l], Evelina La Civita [m], Daniela Terracciano [m, ***], Ferdinando Febbraio [n, 2, **], Amelia Cimmino [a, 2, *]**

[a] Institute of Genetics and Biophysics "A. Buzzati Traverso", National Research Council (CNR), Naples, Italy
[b] Immunology Research Area, B-cell development Unit, Children Hospital Bambino Gesù, Rome, Italy
[c] IRCCS Humanitas Clinical and Research Center, Rozzano - Milan, Italy
[d] Division of Urology, European Institute of Oncology, Milan, Italy
[e] Department of Science and Technology, University of Naples Parthenope, Naples, Italy
[f] Uro-Gynecological Department, Istituto Nazionale per lo Studio e la Cura dei Tumori, Fondazione "G. Pascale"-IRCCS, Naples, Italy
[g] Pathology Unit, Istituto Nazionale per lo Studio e la Cura dei Tumori, Fondazione "G. Pascale"-IRCCS, Naples, Italy
[h] Department of Physical and Mental Health and Preventive Medicine, Section of Pathology, Università degli studi della Campania "Luigi Vanvitelli", Naples, Italy
[i] Institute for Applied Mathematics "Mauro Picone", National Research Council (CNR), Naples, Italy
[l] Translational Molecular Pathology Department and Leukemia Department, University of Texas MD Anderson Cancer Center, Houston, TX, USA
[m] Department of Translational Medical Sciences, University of Naples "Federico II," Naples, Italy
[n] Institute of Biochemistry and Cell Biology, National Research Council (CNR), Naples, Italy

## Supplementary figures

**Figure S1.** A) Violin plot showing uc.8+ intensity score values across normal surrounding urothelium (NSU), low grade (LG) and high grade (HG) tissues. B) Frequency plot showing the scaled distribution of uc.8+ intensity in NSU, LG and HG according to the uc.8+ intensity. ** p-values <0.01.

**Figure S2. Violin and Frequency plots showing the correlation between the uc.8+ intensity score and clinical traits of patients cohort.** (A) Association with sex, (B) age of pts, (C) metastasis and (D) therapy.

**Figure S3. Predicted secondary structure representation of the whole uc.8+.** Sequences associated with the predicted nuclear (light red) and cytosolic (light blue) localization. In purple the overlapped sequences of both localizations.

**Figure S4. uc.8+ intensity of sample belonging to HG and LG, with respect to the cellular localization.** Boxplots describing the relationship between intensity level of uc.8+ (Y axis) and cellular localization (X axis) in BlCa tissues.

**Figure S5.** Fluorescence microscopy of J82 bladder cancer control cells (first lane, Mock) after transfection with PNA-TO scramble-R8 (second lane, scramble PNA) or with TO-PNA1-R8, complementary to uc.8+ sequence (third lane, uc.8+ PNA). Images were recorded with excitation wavelength (lex)=450–490 nm (DAPI) or lex=510–540 nm (PNA-TO); the superimposition of the images recorded is also reported (Merge). Scale bar, 100 mm. Nuclei of J82 cells were stained with DAPI (blue).

## Supplementary Table

**Table S1. Subcellular localization of uc.8+ in BlCa patients.** Data were reported with respect to the lncRNA intensity expression and patients tumor Grade.

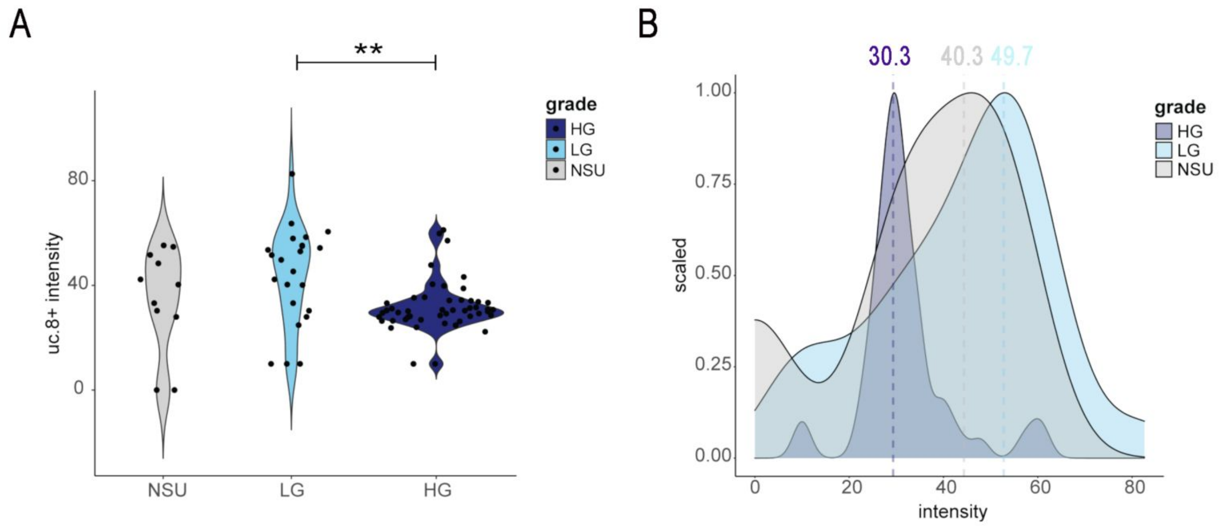


**Figure S1.** A) Violin plot showing uc.8+ intensity score values across normal surrounding urothelium (NSU), low grade (LG) and high grade (HG) tissues. B) Frequency plot showing the scaled distribution of uc.8+ intensity in NSU, LG and HG according to the uc.8+ intensity. ** p-values <0.01.

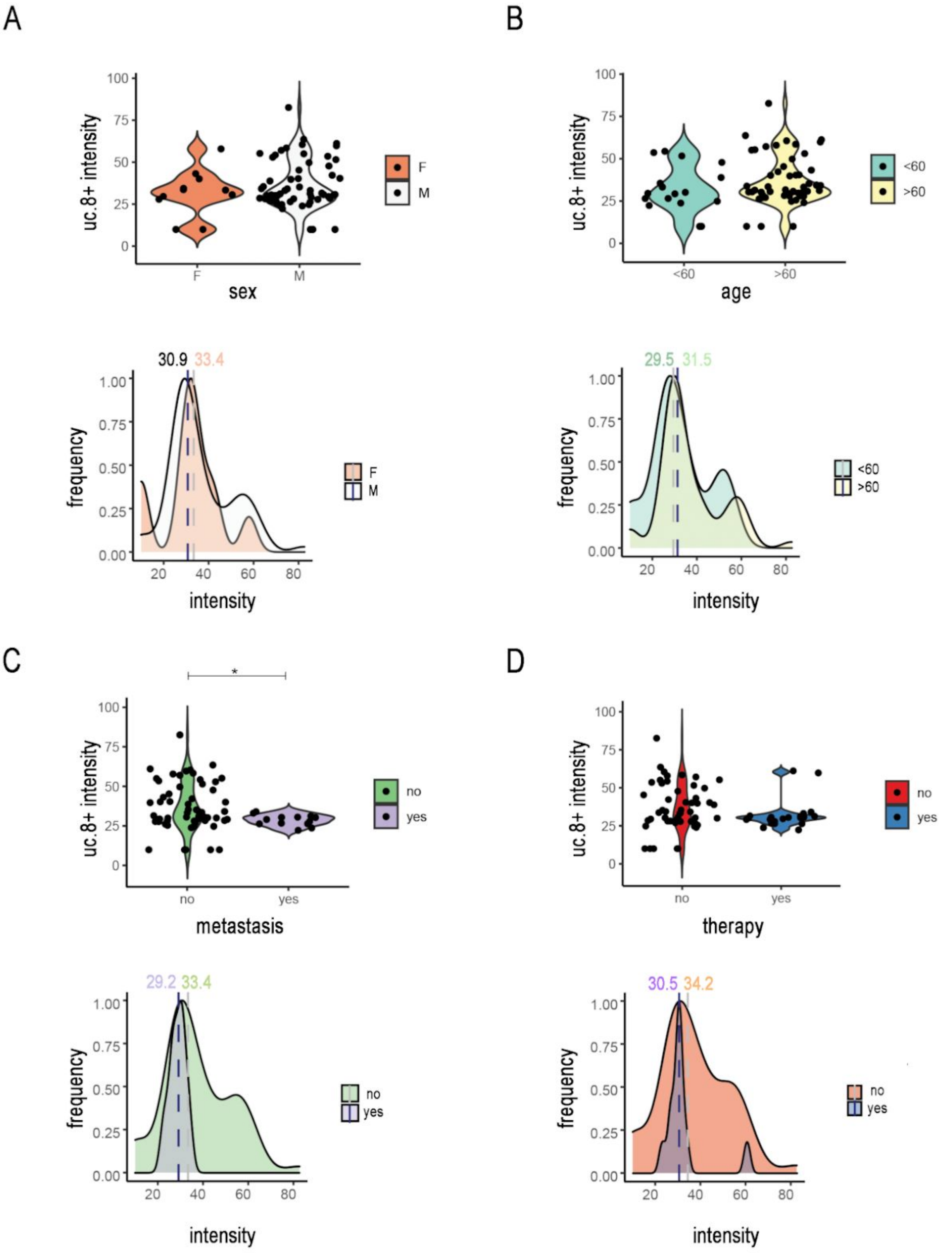


**Figure S2. Violin and Frequency plots showing the correlation between the uc.8+ intensity score and clinical traits of patients cohort.** (A) Association with sex, (B) age of pts, (C) metastasis and (D) therapy.

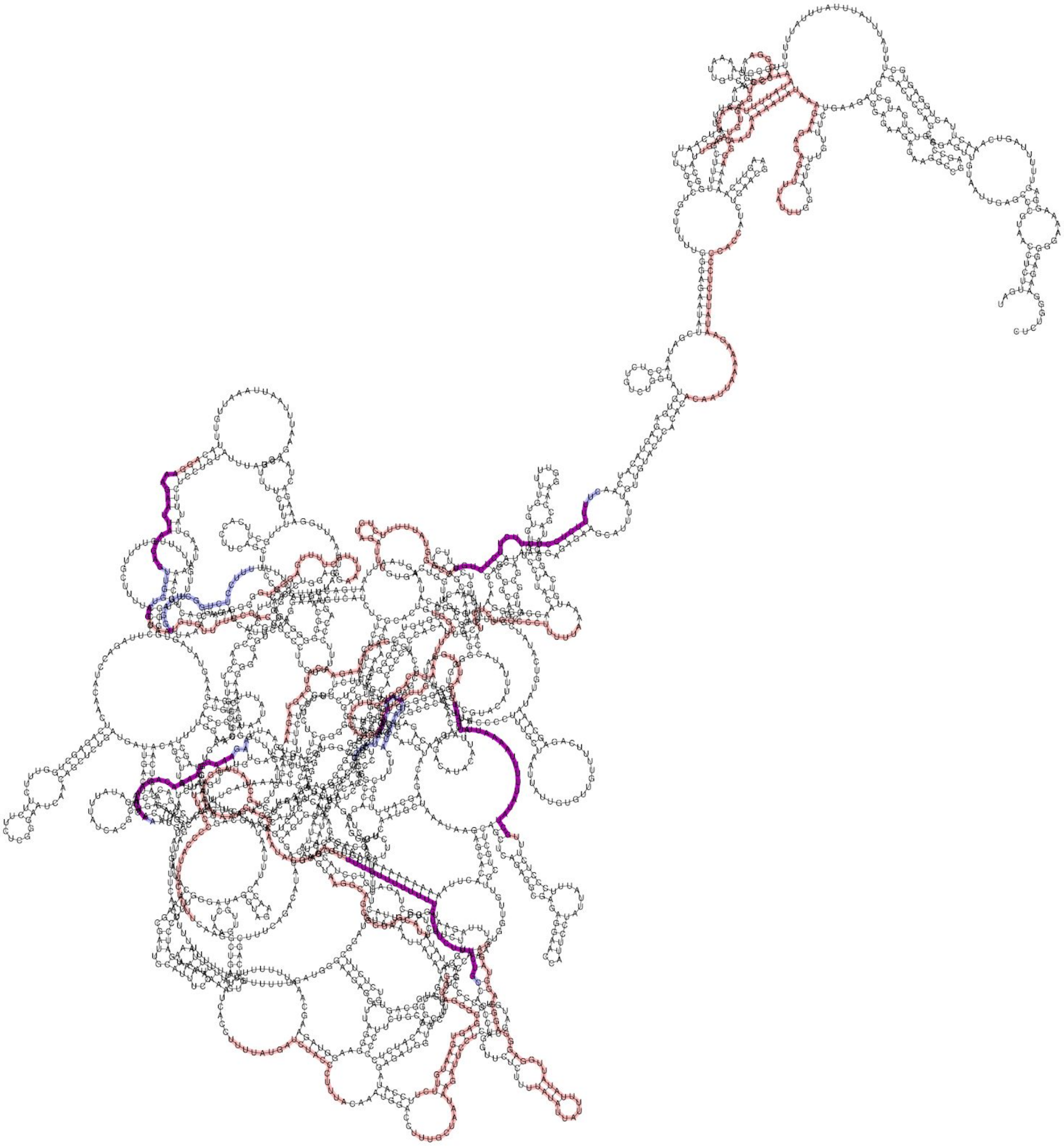

**Figure S3. Predicted secondary structure representation of the whole uc.8+.** Sequences associated with the predicted nuclear (light red) and cytosolic (light blue) localization. In purple the overlapped sequences of both localizations.

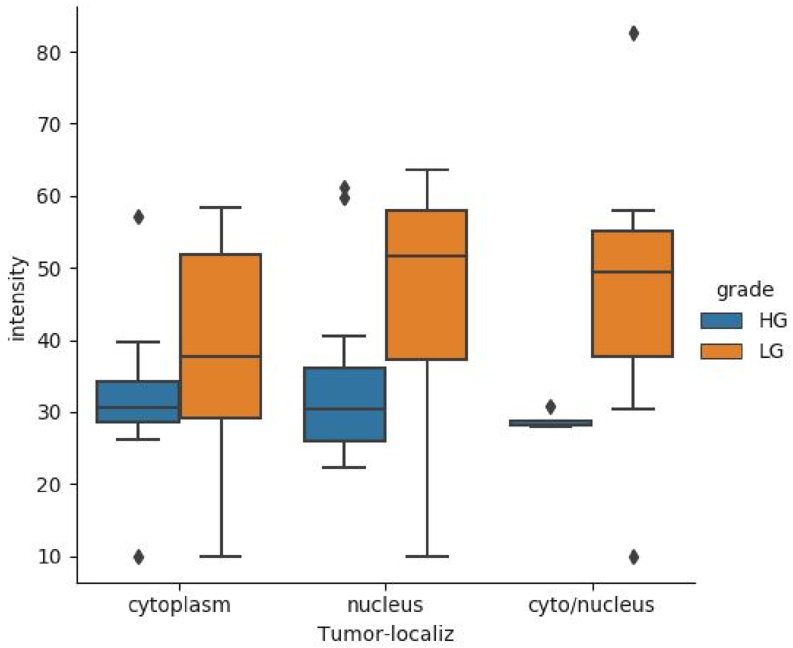


**Figure S4. uc.8+ intensity of sample belonging to HG and LG, with respect to the cellular localization.** Boxplots describing the relationship between intensity level of uc.8+ (Y axis) and cellular localization (X axis) in BlCa tissues.

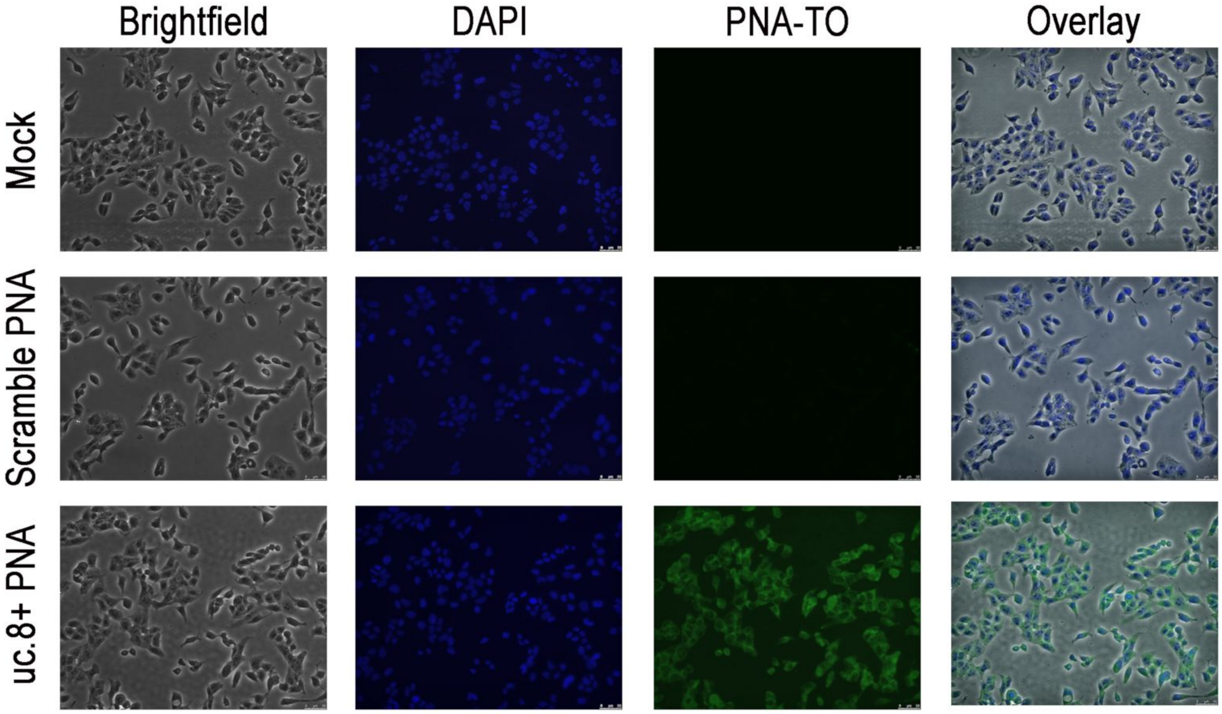


**Figure S5.** Fluorescence microscopy of J82 bladder cancer control cells (first lane, Mock) after transfection with PNA-TO scramble-R8 (second lane, scramble PNA) or with TO-PNA1-R8, complementary to uc.8+ sequence (third lane, uc.8+ PNA). Images were recorded with excitation wavelength (lex)=450–490 nm (DAPI) or lex=510–540 nm (PNA-TO); the superimposition of the images recorded is also reported (Merge). Scale bar, 100 mm. Nuclei of J82 cells were stained with DAPI (blue).

**Table S1. Subcellular localization of uc.8+ in BlCa patients.** Data were reported with respect to the lncRNA intensity expression and patients tumor Grade.

| | % of BlCa patients | | | |
|---|---|---|---|---|
| | Grade | | Intensity-score subgroups | |
| | LG | HG | HI | LI |
| Localization | | | | |
| cytoplasm | 26 | 46 | 26 | 49 |
| nucleus | 30 | 38 | 37 | 35 |
| nucleus-cytoplasm | 44 | 16 | 37 | 16 |
